# Using Existing Indicators to Bridge the Exposure Data Gap: A Novel Natural Hazard Assessment

**DOI:** 10.3390/su162310778

**Published:** 2024-12-09

**Authors:** Adam K. Williams, James K. Summers, Linda C. Harwell

**Affiliations:** 1 Oak Ridge Associated Universities, 1 Sabine Island Drive, Gulf Breeze, FL 32561, USA; 2 Center for Measurements and Modeling, Office of Research and Development, United States Environmental Protection Agency, Gulf Breeze, FL 32561, USA

**Keywords:** resilience, sustainability, CRSI, natural hazard, climate change

## Abstract

Extreme natural hazard events are increasing across the globe, compelling increased climate research on resiliency. Research concerning issues as integrative as climate change and natural hazard resiliency often requires complex methodologies to account for cumulative influences. Indicators can be used to parse complex data to assess the intersection of inputs and outcomes (i.e., cumulative impacts). The Climate Resilience Screening Index (CRSI) is a good example of an indicator framework as it integrates indicators and their associated metrics into five domains (e.g., natural environment, society, and risk), enabling the index to accommodate a variety of inputs in its assessment of resilience. Indicator research, however, is generally limited by the availability of pertinent data. Natural hazard data concerning exposure, loss, and risk are routinely collected by the Federal Emergency Management Agency (FEMA) to create and update the National Risk Index (NRI), a composite index. The NRI can be disaggregated to obtain individual underlying metrics about natural hazard exposure. Quantifying natural hazard exposure requires extensive computation, with each hazard type requiring multiple modifying considerations, such as meteorological adjustments made by subject matter experts. Commonly available natural hazard exposure data, like that from FEMA, combines the spatial extent of historical natural hazard events and the determined value of the affected area. Exposure-related data were retrieved from the National Risk Index and used to create a new composite value to represent only the spatial extent of natural hazard events. Utilizing this new methodology to represent natural hazard exposure alleviates the burden of complex computation. It allows exposure data to be more expeditiously integrated into research and indices relating to natural hazards.

## 1. Introduction

Analyzing complex multivariant data requires a robust and intricate approach. Indicators are commonly used to integrate pertinent multivariant metric data into indices [1]. Using indicators in complex analyses allows researchers to harmonize their understanding of cumulative impacts and outcomes. Indicators, however, are only as useful as the accuracy and timeliness of the data used to compute them. Indicator research has produced useful indices concerning natural hazard risk in recent years. Notable examples of these indices include the Community Resilience Screening Index (CRSI) [1], the Natural Hazards Index v2.0 from the National Center for Disaster Preparedness [2], the Coastal Vulnerability Index from USGS [3], and the U.S. Climate Vulnerability Index produced in partnership by the Environmental Defense Fund, Darkhorse Analytics, and Texas A&M University [4]. While these indices are robust, they aim to ascertain ‘who’ is vulnerable to ‘what’, with minimal focus on the ‘where’ and ‘why’. The ‘why’ refers to meteorological inputs that determine the intensity and frequency of natural hazard events (i.e., climate change), and the ‘where’, or location of a natural hazard occurrence, indicates the geographical extent of exposure.

The observed frequency and severity of natural disasters have increased over the last two decades, with many events reaching unprecedented levels [5]. Globally, urban planning and development efforts have increasingly focused on improving the resilience of built and, to a lesser degree, natural environments to the effects of climate hazards [6]. The severity of impacts from extreme climate hazards is not conditional on the character of the hazard event alone but also on the exposure and vulnerability of the community affected [7]. Several factors influence how natural hazards affect communities (e.g., magnitude, frequency, and preparedness). Arguably, few are as important as recognizing the patterns of potential exposure to these hazards since where and to what extent an adverse event may occur informs the trajectory of building resilience capacity [8]. These factors include natural climate variability, anthropogenic stressors (e.g., land-use change and urbanization), climate change, and socioeconomic status. Adaptive climate risk management, or the adaptation to extreme climate change, focuses on assessing exposure and reducing vulnerability to hazard events while increasing resilience to possible adverse impacts of extreme hazard events [9].

Natural hazards can damage and destabilize community infrastructure, economy, and social service networks, either directly or indirectly, by destroying the natural environment on which communities depend [1]. Climate risk mapping has become a rapidly growing part of the broader climate analytics industry [10]. As impacts of natural hazard events (i.e., infrastructure damage and economic loss) have grown in recent years, there has been an increase in demand for current and accessible data that allow for the analysis of and preparations for future hazard events [11]. Access to hazard data enables the analysis to assess the potential risk of exposure to and impact of natural disasters, such as flooding, earthquakes, wildfires, and hurricanes [12]. The current literature shows a keen interest in developing natural hazard assessment tools that often take shape as indicator frameworks using available hazard data to make communicating risk to the public more specific, feasible, and actionable [13].

The natural environment is a crucial part of sustainability and resilience research, often weighted with consideration for ecological change (e.g., habitation and agriculture) and represented by a dollar amount [14]. This method of weighting the natural environment is useful when assessing economic impacts; however, when discerning the geographical extent of natural hazard exposure on the natural environment, more exclusive measures are needed, such as temporal and spatial scales [8]. They relate natural hazard occurrence to an area-weighted (e.g., developed, agriculture) natural environment domain that provides a basis for determining the extent of the impact rather than the extent of exposure. Concerning exposure, areas historically exposed to extreme natural hazard events regularly have developed a certain level of resilience to those hazards [15]. In areas in which extreme natural hazard events seldom occur, efforts to bolster resilience are less directed, highlighting the need for a method of determining possible modifiers to adverse outcomes [16]. Separating exposure from impact when discussing resiliency enables modifying inputs to more accurately designate consequential aspects of natural hazard events in specific areas [8].

An example of separating exposure and impact is demonstrated in the Climate Resilience Screening Index (CRSI), which was developed by the EPA in 2017 [1] and revised in 2020 [17]. The CRSI provides communities with a tool they can use to ascertain where and what factors negatively impact their resilience to natural hazards. The index has five domains: risk, governance, society, built environment, and natural environment. The risk domain contains two indicators: exposure and loss. Exposure represents the proportion of area exposed to a given natural hazard. Loss represents the historical loss to population, developed area, and agriculture. The ‘exposure’ indicator is further delineated by hazard type, including hurricanes, tornados, coastal floods, drought, earthquakes, wildfires, high-temperature events, inland flooding, landslides, and low-temperature events.

The Climate Resiliency Screening Index utilizes indicators and sub-indices to determine an overall resilience score for each county in the United States (U.S.). The data used in the CRSI have not been updated since its publication in 2017. However, an update to the natural hazard data was published in 2022 [18], providing more current estimates of natural hazard exposure, potential hot spots, and trends. These updated exposure proportions were never integrated with the CRSI, although they used similar methods of determining exposure.

The currency and accessibility of natural hazard data are crucial to provide prompt hazard assessment and precipitate preparedness. Utilizing current data allows for faster replication of analyses, such as those used in the CRSI, to create the ‘risk’ domain and the ‘exposure’ indicator. Previous methods applied to the CRSI and other indices have used data from various secondary sources, often a different source for each natural hazard type. The data required extensive manipulation and analysis to obtain pertinent values for the indicators. The data are often no longer up to date or unavailable.

This paper proposes a streamlined approach to determining exposure values for natural hazards based on publicly available data from the National Risk Index (NRI). The NRI was constructed by FEMA through collaboration with stakeholders and academic partners [19]. The index contains eighteen natural hazard types with measures of exposure and loss for each hazard type. The NRI uses these measures to calculate a ‘risk score’ for each county in the U.S. According to [11], exposure estimates vary across hazard types. To combat this issue, the development team of the NRI differentiated three ways to define hazard exposure: (1) widespread, (2) susceptible area, or (3) representative area or values. Widespread hazard exposure is applied to hazards that impact large areas or occur anywhere in the defined location (i.e., multiple counties) [11]. Examples of widespread occurrence include wildfires, drought, and hurricanes. Susceptible area hazard exposure is applied to natural hazard types with a defined area where the hazard type can occur [11]. Hazard types that utilize the susceptible area exposure methodology include coastal flooding, landslides, riverine (inland) flooding, and wildfires. Representative area exposure is applied to hazard types with a historic event occurrence area [11]. Tornado exposure utilizes this method to determine the area impacted. Expected annual loss (EAL) is another NRI metric, representing the average annual economic loss resulting from natural hazards [19]. EAL is a composite value, combining annualized frequency, the extent of exposure, and the historical loss ratio [20]. The composite indicator, EAL, provided by the NRI incorporates land-use weighting applied to its exposure values to assign a dollar amount to exposure. The conceptual structure of the EAL calculation illustrates the utility of composite indicators. The risk domain in the CRSI employs a conceptual framework similar to the NRI’s EAL.

A novel approach is needed to adequately accommodate the gaps in the availability and currency of natural hazard data. Utilizing the data processed and published by the NRI will produce accurate composite exposure values (i.e., extent, not impact) and allow faster replication and modernization of new and existing indices.

## 2. Methods

To provide current and accessible climate hazard exposure data for future applications, including updates to the risk domain of the CRSI, a method of factoring available data obtained from the NRI, was developed. Factoring, or factor analysis, simplifies complex variables by multiplication or division, allowing the examination of the underlying relationship between the variables [21]. Exposure data measuring the proportion of area exposed to a climate hazard event and annualized frequency (AF) data measuring the average count of exposures for a specific area (i.e., county area) annually are available from the NRI [19]. These data are extracted from the NRI dataset and are used to create a new composite value to represent the exposure potential of a geographical area.

### 2.1. Software and Data Processing

Data were collected from a readily available secondary source to create a new composite value for county hazard exposure. The National Risk Index (NRI) provides the annualized frequency of climate hazard exposures—calculated as events per year or event days per year—and exposure area. The exposed area and the annualized count of hazard events were extracted from the NRI’s dataset. The NRI dataset contains 18 natural hazard types displayed in Table 1 [19]. The NRI indicators extracted and used in this analysis are annualized frequency (AF), impacted area square miles (sq. mi), and area (county area). The dataset was compiled in a Microsoft Excel workbook for manipulation, analysis, and visualization in ArcGIS Pro version 3.2.1 [22].

### 2.2. Factor Equation

To maintain the objectivity of exposed area estimates used in indices such as the CRSI, while using data obtained from the NRI, a simple and intuitive method was created to provide an accurate value for illustrating natural hazard exposure. This paper provides an updated, consistent method for transforming the NRI’s annualized frequencies of natural hazards and the area of counties impacted by each hazard to combat the issue of accessing and processing hazard data from multiple sources. The annualized frequencies and impacted area values are used to create the new composite value (NCV), providing researchers with an intuitive metric to integrate into or update indicator frameworks. The new composite value is provided in the following equation:

$$\text{NCV}=\text{Annualized}\;\text{frequency}\times \frac{\text{Impacted}\;\text{area}}{\text{Total}\;\text{area}}$$

where

NCV = the new composite value;

Annualized frequency (AF) = the average annual count of exposures for U.S. counties; Impacted area = the total area of the county exposed to a climate hazard event;

Total area = the area of the geographical reference and analysis (i.e., census tract and county).

All calculations were processed in Excel before being returned to ArcGIS Pro for visualization.

Spatial processing of hazard data was executed in ArcGIS Pro version 3.2.1.

### 2.3. Processing of NRI Data Utilizing New Factor Method

The downloadable NRI [19] dataset contains the area of exposure and annualized frequency used to calculate the NRI composite index for a specific spatial resolution (e.g., county). Using area exposed values of the NRI, the proportion of area exposed was calculated for each natural hazard. The proportion of county area exposed was multiplied by the annualized frequency of the hazards affecting the county to produce a modified exposure factor. Each exposure factor for each hazard type was normalized using a simple minimum–maximum approach (min–max) [23] to put all hazards on the same scale and create a new composite value. These normalized values were summed to produce an overall exposure risk value, demonstrating the combined hazard exposure for all nine natural hazards. Again, these values were normalized via min–max to produce an overall exposure score for each county. The newly calculated NCV was geospatially visualized and compared to the original data. The normalized composite exposure values were assigned to a graduated symbology with 5 classes using the equal interval method (Data Classification Methods ArcGIS Pro) to visualize the results for each natural hazard. For comparison purposes, the proportion of area exposed, calculated from the NRI data, was then normalized to the same scale as the NCV.

## 3. Results

Area exposed estimates provided by the NRI dataset appear to be distributed asymmetrically, more toward “fully exposed” for some of the natural hazards than the NCV estimates. For simplicity, the term “possible determinacy” will be used to generalize the NRI’s three methodologies to estimate hazard exposure (i.e., widespread, susceptible area, and representative area).

**Figure 1.**
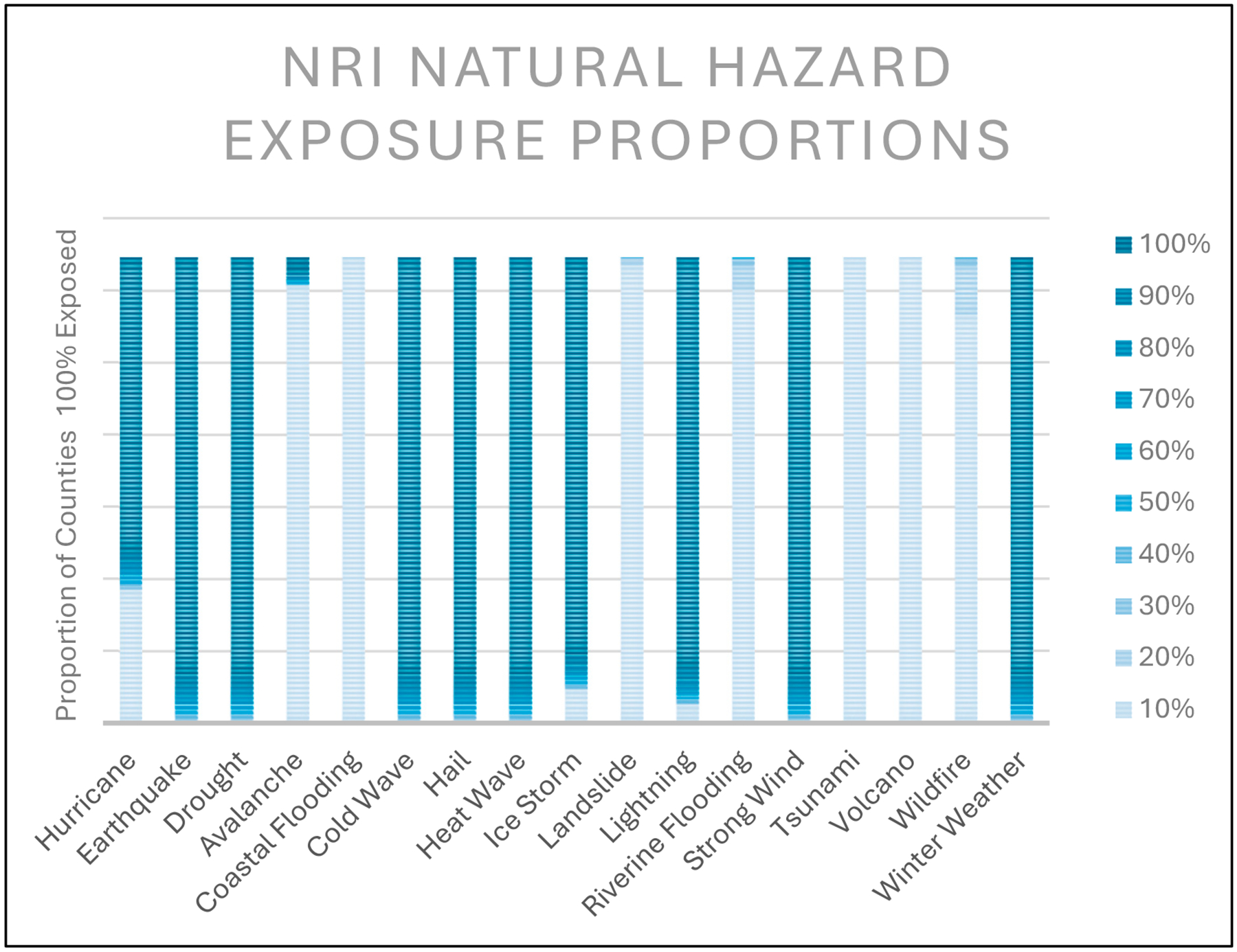
A bar chart showing the percentage of counties with 100% exposure to each of the eighteen natural hazard types using the NRI exposure parameters without adjustment.

To further investigate this possible discrepancy, the NRI “Exposure—Impacted Area” (sq mi) values for 17 of the natural hazard types were extracted to calculate the percentage of the area impacted for each county (Figure 1). Ten of the seventeen natural hazards appear to have the majority of counties labeled as 100% exposed: hurricanes, earthquake, drought, cold wave, hail, heat wave, ice storm, lightning, strong wind, and winter weather. Hurricane exposure differs from other hazards as many counties are labeled with zero exposure. Ice storms and lightning also have fewer counties labeled as 100% exposed. Earthquake, drought, cold wave, hail, heat wave, strong wind, and winter weather all appear to have similar exposure estimates, with a positive skew towards 100% exposure.

**Figure 2.**
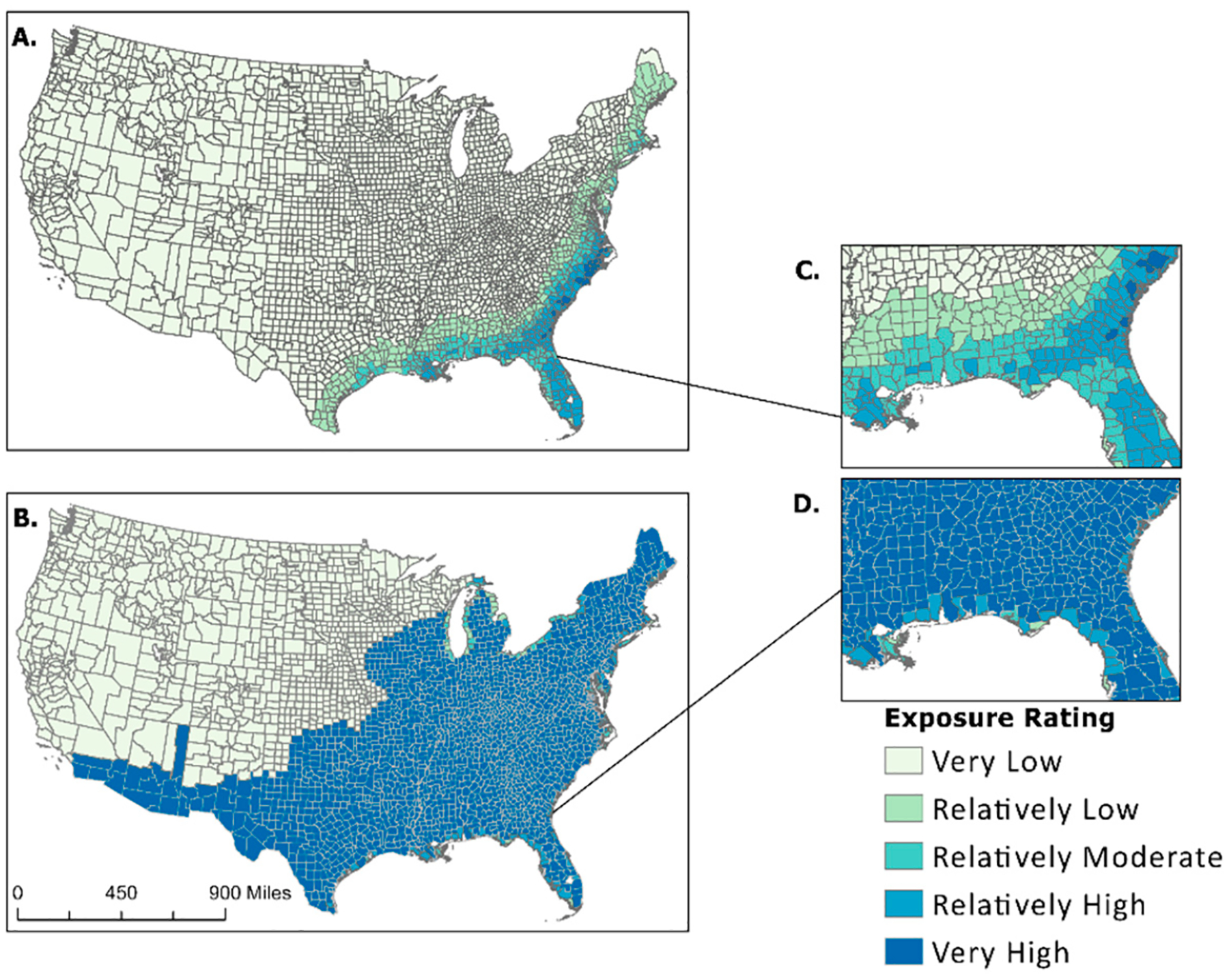
A comparison of NCV (**A**,**C**) applied to NRI attributes and NRI raw percentages (**B**,**D**) showing ratings of county areas exposed to hurricanes.

The NRI’s use of varying exposure definitions could explain the skewness seen in the dataset for drought, earthquake, strong wind, tornado, and hurricane. It is stated within the NRI Technical Documentation (https://hazards.fema.gov/nri/Content/StaticDocuments/DataDownload/Archive/v117_0/fema_national-risk-index_technical-documentation.pdf (accessed on 5 January 2024) that subject matter experts are consulted on possible areas of occurrence for each hazard type. According to these experts, all counties could be exposed to drought, tornados, and earthquakes due to widespread hazard exposure. Hurricanes were also deemed widespread but only to the historical extent of exposure, which included the entire East Coast, Midwest, Texas, and southern counties in New Mexico, Arizona, and California. The proportion of county area, calculated from the NRI, was normalized to the same scale as the NCV. A visual comparison between the NRI percent area exposed and the NCV for hurricanes is shown in Figure 2. The maps in Figure 2 depict the difference in percent area exposed from the NCV calculations (Figure 2A,C) and values obtained from the NRI (Figure 2B,D). The results show all coastal counties (on the East Coast) had lower exposures from hurricanes than inland counties, with most inland counties labeled as 100% exposed.

**Figure 3.**
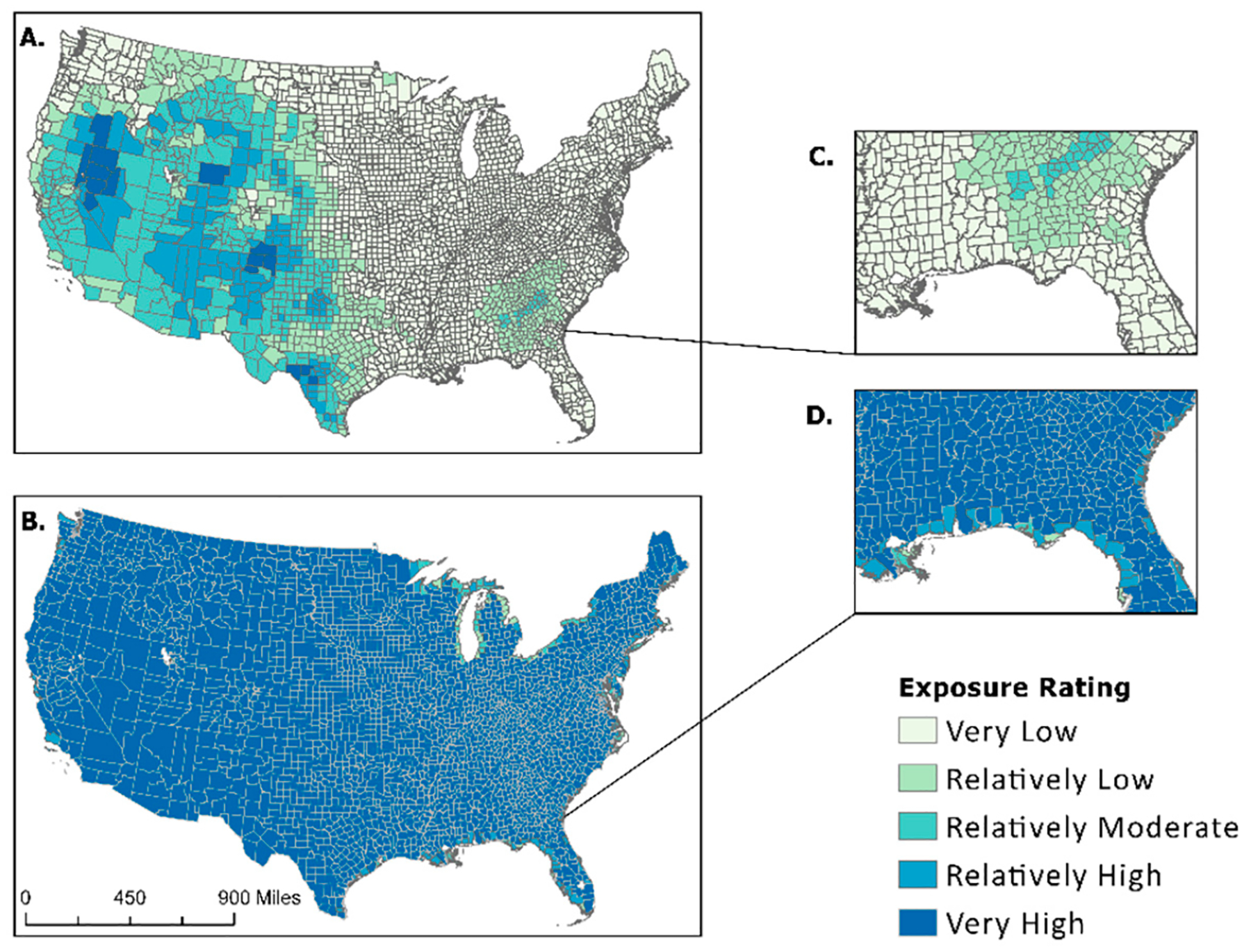
A comparison of NCV (**A**,**C**) applied to NRI attributes and NRI raw percentages (**B**,**D**) showing ratings of county areas exposed to drought.

Drought percent area exposed values obtained from the NRI designate all inland counties as 100% exposed, and coastal counties have varying but high exposure percentages. The NRI uses a widespread occurrence methodology to determine drought exposure for the U.S. Although it may be true that every county experiences drought conditions to some extent, the widespread determination of exposure does not allow for more precise examinations. A visual comparison between the NRI percent area exposed and the NCV for drought is shown in Figure 3. The maps in Figure 3 depict the difference in percent area exposed between the NCV calculation (Figure 3A,C) and values derived from the NRI (Figure 3B,D), demonstrating the effect of the NRI’s use of possible determinacy. After taking the proportion of area exposure calculated from the “Exposure—Impacted Area” (sq. mi) provided within the NRI, it is clearly shown that the NRI has deemed all inland states to have very high exposure ratings and varying exposure ratings for coastal counties.

**Figure 4.**
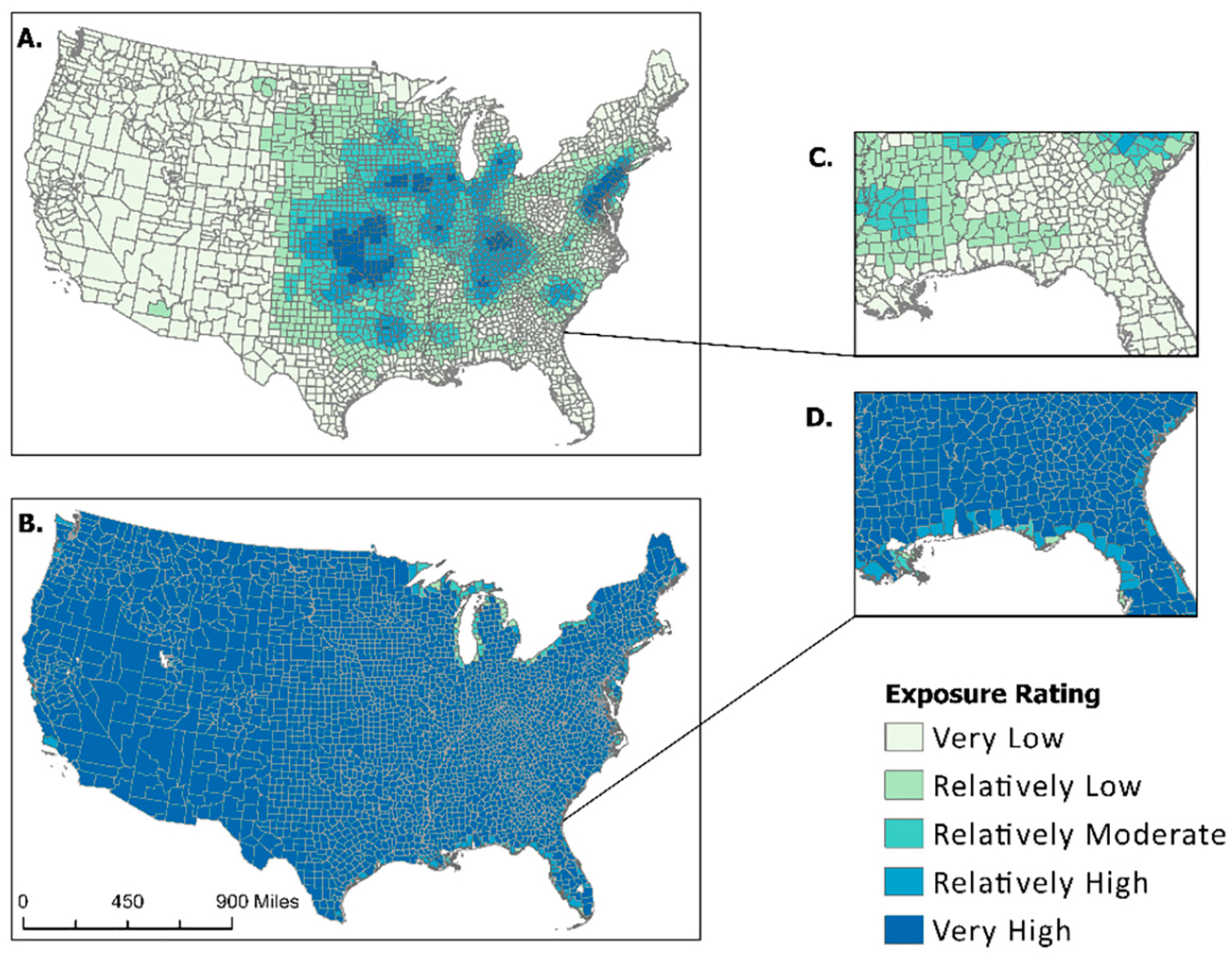
A comparison of NCV (**A**,**C**) applied to NRI attributes and NRI raw percentages (**B**,**D**) showing ratings of county areas exposed to strong wind.

Strong wind is another natural hazard for which the NRI has employed possible determinacy. As shown in Figure 1, strong wind is one of nine natural hazards for which nearly all counties are deemed as fully exposed. A visual comparison between the NRI percent area exposed and the NCV for strong wind is shown in Figure 4. This map depicts the drastic difference in the percent area exposed between the NCV calculation (Figure 4A,C) and values derived from the NRI (Figure 4B,D). A concentration of strong wind exposures can be seen in the Midwest on the NCV map (Figure 4A,C), while the map produced by the NRI (Figure 4B,D) shows nearly all inland counties as fully exposed.

**Figure 5.**
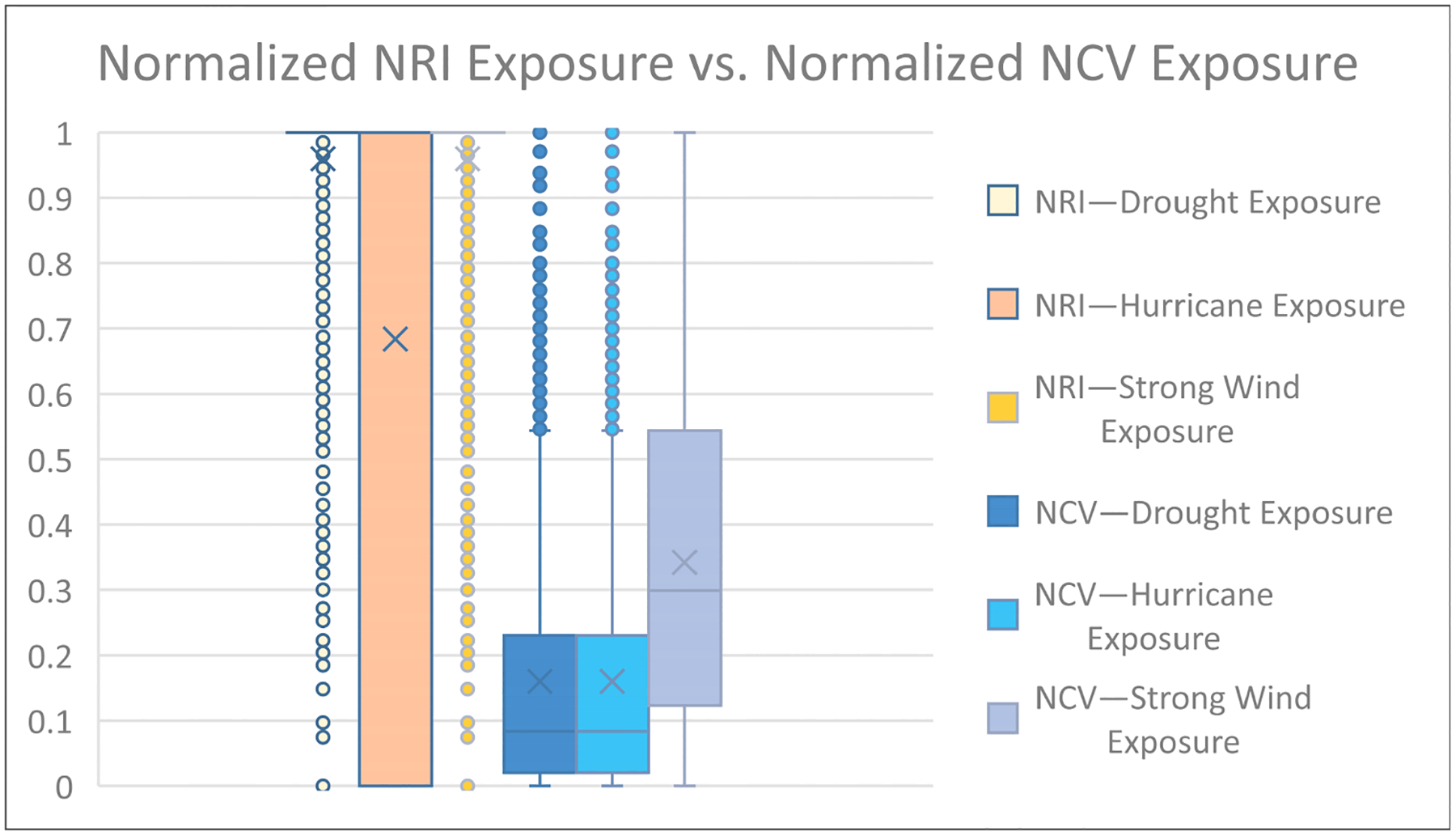
A box-and-whisker plot showing the distribution of exposure estimates for normalized percent area calculated from the NRI and the NCV calculated from NRI data.

A box-and-whisker plot further illustrates that the NCV transformation yields a more even distribution of exposure values (Figure 5). NRI exposure estimates were obtained by normalizing calculated percent area using the same method as the NCV normalization stated above. This data normalization allows for NRI and NCV comparisons. The first three series from the left in Figure 5 represent the normalized percent area data calculated from the NRI. “NRI—Drought Exposure” and “NRI—Strong Wind Exposure” appear skewed, with their median nearly reaching the maximum value represented. Hurricane exposure estimates from the NRI percent area appear to fall into a single category: fully exposed. However, the lower tail of the box-and-whisker plot for “NRI—Hurricane Exposure” cannot be seen. This is due to the first quartile of hurricane exposure estimates being zero, thus stretching the box (i.e., Quartile 1–Quartile 3) from zero to one. This leaves all those counties receiving zeros for their exposure estimates off the plot. The last three series on the plot represent exposure estimates produced by the NCV methodologies stated above. In these series, NCV hurricanes and droughts appear to have a more even distribution of values, while strong winds represent a higher mean of exposure (Figure 5).

**Figure 6.**
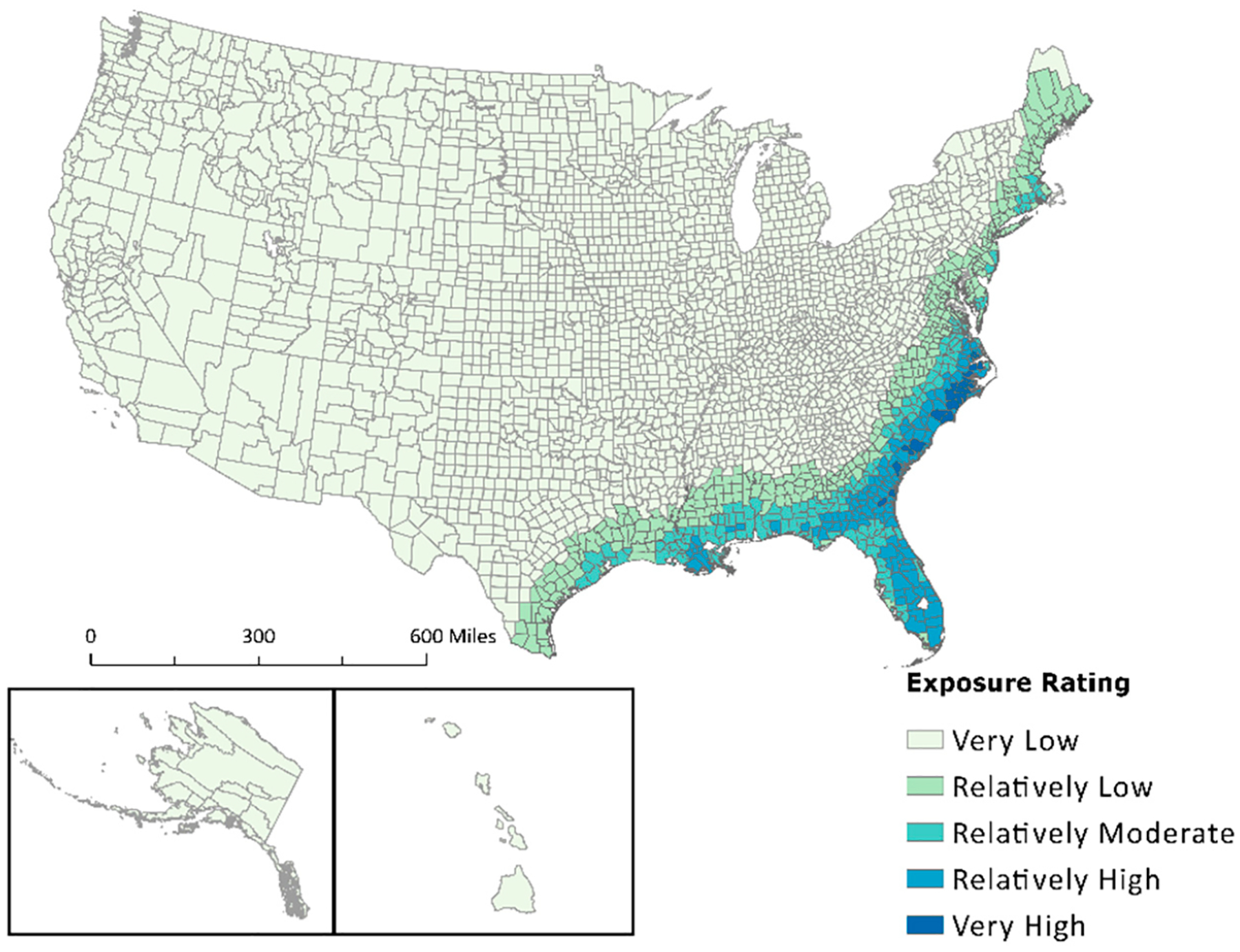
Spatial analysis of hurricane data showing hazard exposure ratings retrieved from the NRI.

**Figure 7.**
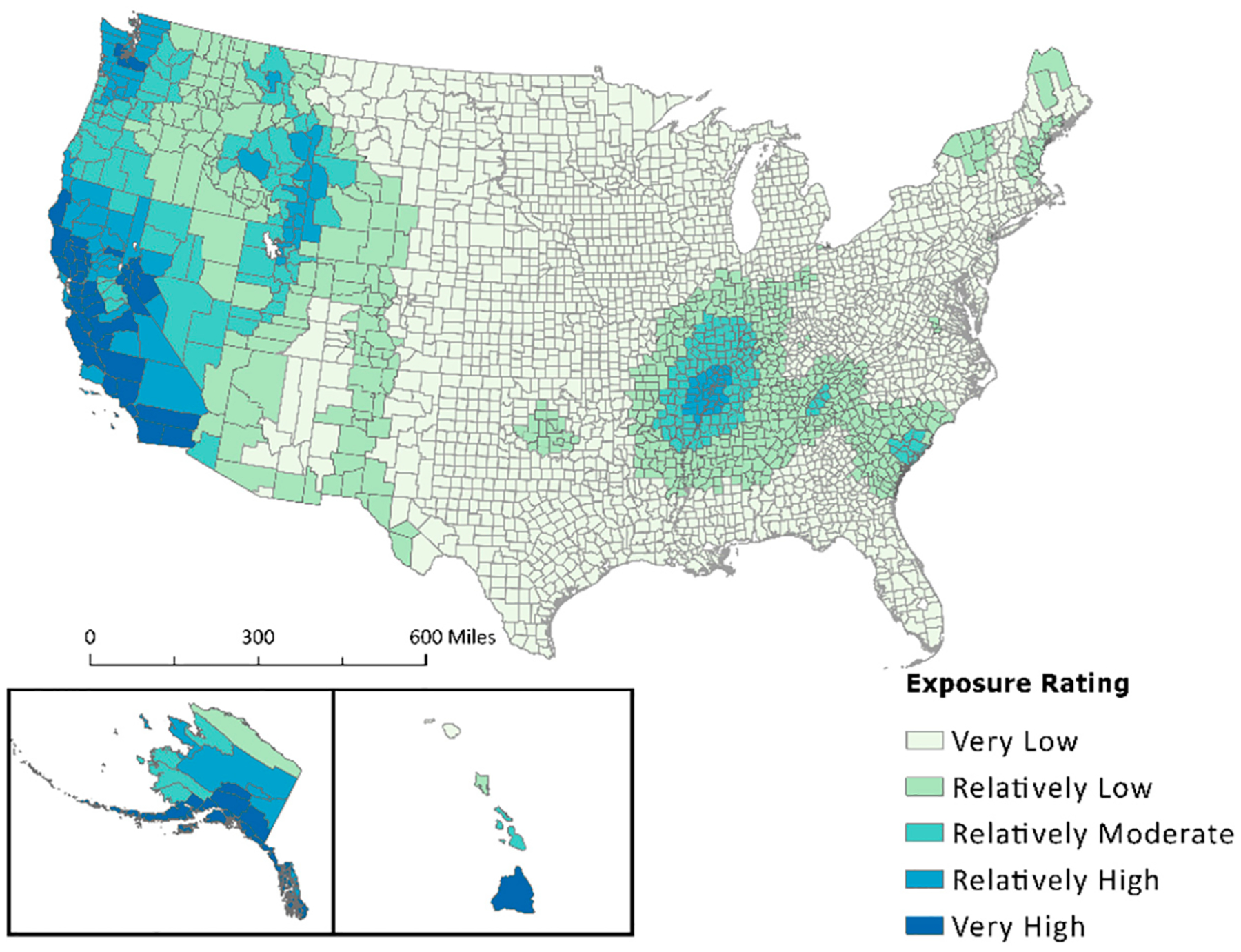
Spatial analysis of earthquake data showing hazard exposure ratings retrieved from the NRI.

**Figure 8.**
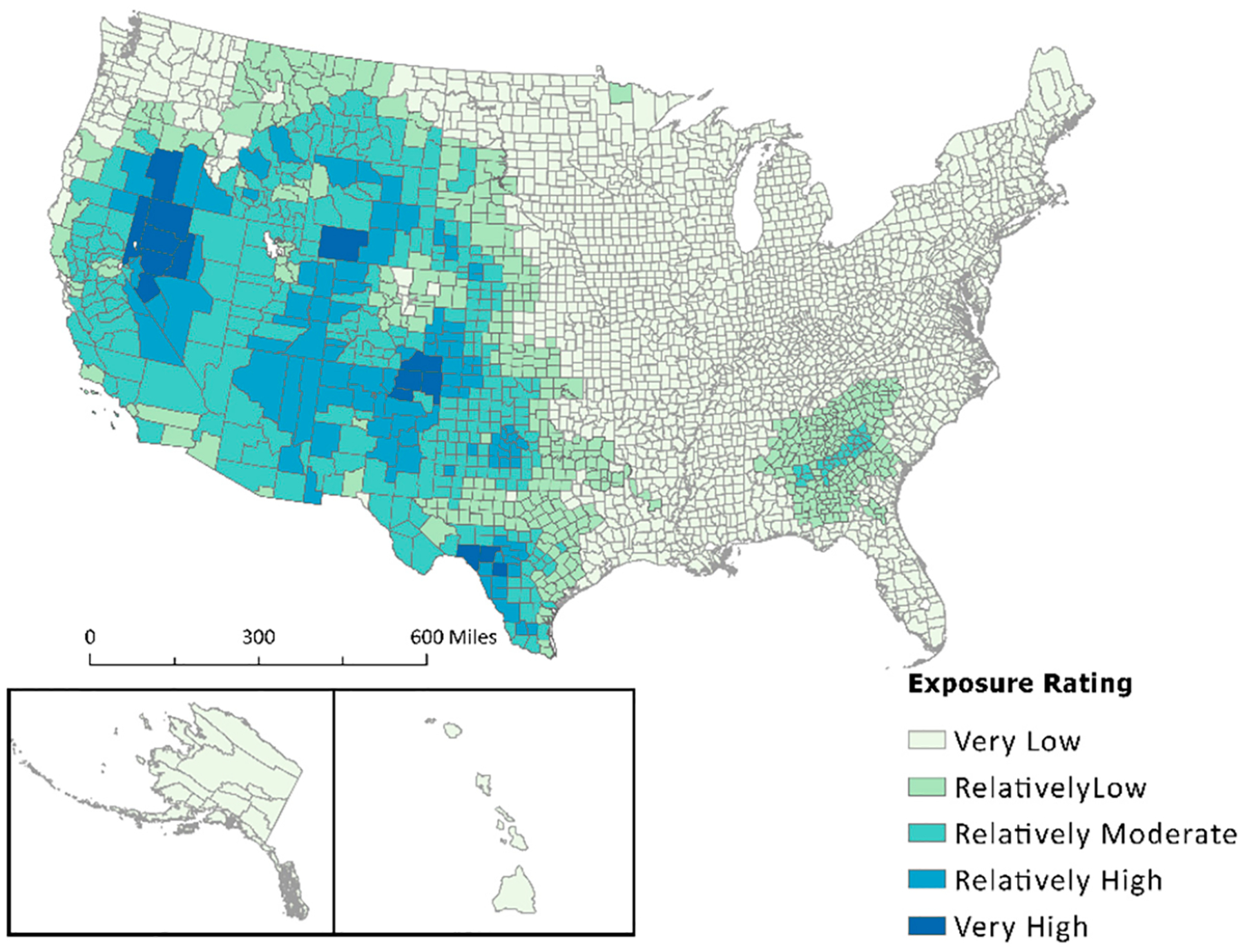
Spatial analysis of drought data showing hazard exposure ratings retrieved from the NRI.

Figures 6–8 detail county NVC ratings of climate hazard exposures for hurricanes, earthquakes, and drought based on the NRI data.

U.S. hurricane event exposure is depicted with a graduated symbology representing an exposure rating between ‘Very Low’ and ‘Very High’ (Figure 6). Southeastern states show the highest level of hurricane exposure, continuing up the eastern coast into northeastern states. Louisiana, Florida, Georgia, South Carolina, and North Carolina have the highest hazard exposure ratings for hurricanes. The extent of exposure wanes as proximity to the coast decreases, i.e., as one travels inland. States on the West Coast received ‘Very Low’ ratings for hazard exposure, with two notable exceptions in southern Arizona receiving a ‘Moderately Low’ rating. Multiple counties in southern California and Arizona received higher exposure values before applying the new factoring method. Alaska and Hawaii received a ‘Very Low’ exposure rating.

County-level earthquake hazard exposure ratings for the U.S. are shown in Figure 7. Western states, including California, Oregon, Utah, Montana, Wyoming, and Washington, received higher earthquake exposure ratings. ‘Very High’ exposure ratings appear centralized in California and Washington State. Arizona and Nevada are shown to have a relatively high exposure rating. Alaskan counties are assigned ratings from ‘Relatively Low’ to ‘Very High,’ with the higher-rated counties located to the south. The Hawaii islands have earthquake exposure ratings from ‘Very Low’ to ‘Very High’. Hawai’i County, the biggest island, shows a ‘Very High’ exposure rating, with each following island county receiving lower ratings the further it is from Hawai’i County. The Midwest also shows higher exposure ratings in Arkansas, Tennessee, Missouri, Illinois, and Kentucky. South Carolina and northern Georgia received ‘Relatively Low’ to ‘Relatively Moderate’ ratings. Earthquake exposure ratings decrease as proximity to the event source decreases.

Spatial distributions of drought exposure ratings are shown in Figure 8. There is a concentration of exposure in the western half of the U.S., with parts of Oregon, California, Wyoming, Utah, Colorado, New Mexico, and Texas receiving a ‘Very High’ exposure rating. A cluster of ‘Very High’ exposure ratings stretches from Oregon into Nevada. On the East Coast, drought exposure is far less, with most of the eastern U.S. receiving ‘Very Low’ exposure ratings. There is, however, a concentration of ‘Relatively Low’ and ‘Relatively Moderate’ exposure ratings affecting most of Georgia and parts of Florida, Alabama, South Carolina, North Carolina, and Tennessee.

## 4. Discussion

Indicators are commonly used in evidence-based research to produce accurate analyses and create common methodologies. Indicators are often placed in two groups: qualitative and quantitative. Qualitative indicators can be described as value-based, and measure perception rather than numerical impacts. Quantitative indicators provide measurable data, allowing for the accurate analysis of exposure and impacts [24]. Indicators have enabled researchers to advance their understanding of cumulative impacts and outcomes [25]. However, the data or measures used to create indicators are not always readily available [16]. Availability refers to the content of the data and the time period for which the data represent. Here lies the crux of indicator research: indicators are only as useful as their measures are accessible and temporally current.

The methodologies for quantifying exposed areas used in two previous manuscripts by Summer et al. [1,17] on natural hazards differ from the NRI methodology. Both these manuscripts and the NRI use georeferenced data to determine hazard exposure and impacted areas. However, the NRI’s use of possibility determinacy, such as widespread hazard exposure, contributes to the apparent differences or skewness in exposure area values for half of their hazard types: drought, earthquake, hurricane, tornado, cold wave, hail, heat wave, lightning, ice storm, winter weather, and strong wind (Figure 1). Skewness measures asymmetry within data distribution [26]. The discrepancy between methodologies introduces limitations that do not allow for a direct comparison to Summers et al.’s previous manuscripts [1,17].

Exposure data received from secondary sources and used in both Summers et al.’s previous manuscripts [1,17] and FEMA’s National Risk Index are often associated with a count of events or an annualized frequency. Annualized frequency is the number of events or event days divided by the time period of record. This value is useful because it helps determine which counties are often exposed to certain hazard types. This means annualized frequency can be used to normalize exposure values. Applying this to the artificially inflated “Exposure—Impacted Area” (sq mi) metrics extracted from the NRI allowed for data standardization. Visualizing the difference in exposed area between raw NRI data and the results of the new composite value (Figures 2–4) provided a stark contrast, demonstrating both the effect of possible determinacy used by the NRI and the utility of the NCV proposed in this paper. As seen in the box-and-whisker plot (Figure 5), the raw exposure values from the NRI do not offer normal distributions of estimates, with large sums of counties receiving identical values. Drought and strong wind have a positive skew with their means sitting near the upper limit of exposure estimates, meaning the NRI counts nearly all counties as fully exposed (Figure 5). Despite having nearly all observations fall between Quartile 1 and Quartile 3 (i.e., within the box), hurricane also has a positive skew. However, the NCV exposure estimates appear to have a far more even distribution of estimates for the three selected comparison hazards (Figure 5). Drought and hurricane are both skewed, to a lesser degree than the NRI, but in the negative direction, meaning fewer counties were exposed or the degree of exposure was lower (Figure 5). NCV estimates for strong wind exposure have a distribution that is closest to normal, meaning less skewing and a more even distribution of high to low exposure estimates across the country. The box-and-whisker plot (Figure 5) further illustrates the effect of possible determinacy on exposure estimates independent of georeferencing. Taking a closer look at the NCV results, hurricane (Figure 6), earthquake (Figure 7), and drought (Figure 8) have a graduated symbology. This representation of these natural hazards is proportionate to exposure estimates seen in previous manuscripts (e.g., the CRSI) and comparable to a historic natural hazard event occurrence due to the use of annualized frequency in the NCV.

This method also expedites future iterations of natural hazard research, such as the exposure metric in the CRSI. The NRI will provide updated exposure values and annualized frequencies as they become available from secondary sources, relinquishing the need to process and analyze large amounts of secondary data in-house.

## 5. Conclusions

Indicators, more broadly, indicator research, are only as good as their data. Without accurate and current data, indicator frameworks fail to provide valuable insights into the interconnectedness of potential outcomes. In the realm of resiliency, indicator frameworks provide useful insights concerning communities, especially as they relate to natural hazards. However, the data needed to populate these indicators require much labor as they come from multiple entities and require extensive manipulation to represent natural hazard exposure. Exposed area data for natural hazards offer invaluable information for communities historically affected by natural hazards. However, the amount of data processing necessary to transform raw data into actionable estimations has impeded researchers’ ability to assess community resilience. The lack of access to clean, actionable natural hazard exposure data has far-reaching implications and affects every county in the U.S.

Data were retrieved from the National Risk Index to circumvent this issue. The NRI utilizes its unique exposure estimation methodology developed by subject matter experts. However, the purpose of the NRI is broader than hazard exposure alone, encompassing multiple aspects of risk (e.g., social vulnerability, loss, and resiliency). For this reason, the data retrieved from the NRI must undergo a small transformation to accurately estimate the exposed area for a natural hazard. The method proposed in this paper provides a streamlined approach to estimating natural hazard exposure areas. Utilizing data from the National Risk Index to create a new composite value reduces the computation necessary to obtain exposed area values and allows researchers to produce more expeditious analyses concerning natural hazard preparedness, resilience, and recovery.

## Figures and Tables

**Table 1.** Natural hazard types provided by the NRI.

Included Natural Hazard Types:		
Avalanche; Coastal Flooding; Cold Wave; Drought; Earthquake; Hail;	Heat Wave; Hurricane; Ice Storm; Landslide; Lightning; Riverine Flooding;	Strong Wind; Tornado; Tsunami; Volcanic Activity; Wildfire; Winter Weather.

Data source: The National Risk Index. FEMA.gov. (2023) [19]. https://hazards.fema.gov/nri/ (accessed on 5 January 2024).

## Data Availability

The data are available in a publicly accessible repository [CRSI Data] [Data(\\AA\ORD\GBR)(L:)/Priv/SHC_264/Adam/Nation Risk Index] [].
